# Supplementary material for: The impact of cyberbullying on mental health outcomes amongst university students: A systematic review
Source: PLOS Ment Health. 2024 Nov 13;1(6):e0000166. doi: 10.1371/journal.pmen.0000166 (PMC12798282; doi:10.1371/journal.pmen.0000166)
Supplement: S1 Search strings — (DOCX) [file pmen.0000166.s002.docx]

**Medline:**

(((“Cyberbullying” [MeSH Terms]) OR (“Cyberbullying*”) OR (“Online Harassment”) OR ((“Online Social Networking” [MeSH Terms]) AND (“Harassment, Non-Sexual” [MeSH Terms])) OR (“Online Bullying*”) OR ((“Online Social Networking” [MeSH Terms]) AND (“Bullying” [MeSH Terms])) OR (“Trolling*”) OR (“Troll*”) OR (“Online Trolling*”) OR ((“Social Media*”) AND “Trolling*”)) OR ((“Social Media*”) AND (“Cyberbullying*”)) OR ((“Social Media” [MeSH Terms]) AND (“Cyberbullying” [MeSH Terms])) OR ((“Social Media*”) AND (“Online Harassment*”)) OR ((“Social Media [MeSH Terms]”) AND (“Online Social Networking” [MeSH Terms]) AND (“Harassment, Non-Sexual” [MeSH Terms])) OR ((“Social Media*”) AND (“Online Bullying*”)) OR (“Cyberaggression”) OR (“Cyber-aggression”) OR (“Cyberabuse”)) AND ((“Mental Health” [MeSH Terms]) OR (“Depression*”) OR (“Depression” [MeSH Terms]) OR (“Depressive Disorder*”) OR (“Depressive Disorder” [MeSH Terms]) OR (“Anxiety*”) OR (“Anxiety” [MeSH Terms]) OR (“Suicid*”) OR (“Emotional”) OR (“Mental”) OR (“Psychological”) OR (“Psychopathological”)) AND ((“University*”) OR (“Universities*”) OR (“Universities” [MeSH Terms]) OR (“Undergraduate*”) OR (“Postgraduate”) OR (“College”) OR (“Post-Secondary Education”) OR (“Tertiary Education”))

**Embase:**

(‘Cyberbullying’ OR ‘Cyberbullying’/exp OR ‘Online Harassment’ OR ‘Online Bullying’/exp OR ‘Trolling’ OR ‘Trolling’/exp OR ‘Cyberaggression’ OR ‘Cyber-aggression’ OR ‘Cyberaggression’/exp OR ‘Cyberabuse’) AND (‘Mental Health’ OR ‘Mental Health’/exp OR ‘Depression’ OR ‘Depression’/exp OR ‘Anxiety’ OR ‘Anxiety’/exp OR ‘Suicidal’/exp OR ‘Suicidal’ OR ‘Emotional’/exp OR ‘Emotional’ OR ‘Mental’/exp OR ‘Mental’ OR ‘Psychological’/exp OR ‘Psychological’ OR ‘Psychopathological’/exp OR ‘Psychopathological’) AND (‘University’ OR ‘Universities’ OR ‘Undergraduate’ OR ‘College’ OR ‘Postgraduate’ OR ‘Post-Secondary Education’ OR ‘Tertiary Education’)

**Cochrane:**

(“Cyberbullying” OR “Online Harassment” OR “Online Bullying” OR “Trolling” OR “Troll*” OR “Cyberaggression” OR “Cyber-aggression” OR “Cyberabuse”) AND (“Mental Health” OR “Depression” OR “Anxiety” OR “Emotional” OR “Mental” OR “Psychological” OR “Psychopathological”) AND (“University” OR “Postgraduate” OR “College” OR “Post-secondary Education” OR “Tertiary Education”)
