# Supplementary material for: The impact of cyberbullying on mental health outcomes amongst university students: A systematic review
Source: PLOS Ment Health. 2024 Nov 13;1(6):e0000166. doi: 10.1371/journal.pmen.0000166 (PMC12798282; doi:10.1371/journal.pmen.0000166)
Supplement: S1 Table — (DOCX) [file pmen.0000166.s003.docx]

| **Supplementary Table 1: Quality Assessment of included studies** | | | | | | | | | | | | | | | |
| --- | --- | --- | --- | --- | --- | --- | --- | --- | --- | --- | --- | --- | --- | --- | --- |
| **Study** | **Quality Assessment Criteria** | | | | | | | | | | | | | | **Quality** **Rating** |
|  | **Criteria 1** | **Criteria 2** | **Criteria 3** | **Criteria 4** | **Criteria 5** | **Criteria 6** | **Criteria 7** | **Criteria 8** | **Criteria 9** | **Criteria 10** | **Criteria 11** | **Criteria 12** | **Criteria 13** | **Criteria 14** |  |
| Kraft et al. | Y | Y | N | Y | N | N | N | NA | N | N | N | NA | NA | N | **Poor** |
| Lai et al. | Y | N | CD | Y | N | N | N | NA | N | N | N | NA | NA | N |  |
| Anis-ul-Haque et al. | Y | N | CD | N | N | N | N | NA | Y | N | Y | NA | NA | N | **Fair** |
| Arafa et al. | Y | Y | Y | Y | Y | N | N | NA | Y | N | Y | NA | NA | N |  |
| Beran et al. | Y | N | Y | Y | N | N | N | NA | Y | N | N | NA | NA | Y |  |
| Faucher et al. | Y | Y | CD | Y | N | N | N | NA | Y | N | N | NA | NA | N |  |
| Lee J. | Y | Y | N | Y | Y | N | N | NA | Y | N | Y | NA | NA | N |  |
| Lindsay et al. | Y | N | Y | Y | N | N | N | NA | Y | N | Y | NA | NA | N |  |
| Snaychuk et al. | Y | N | CD | Y | N | N | N | NA | Y | N | Y | NA | NA | N |  |
| Zalaquett et al. | Y | N | CD | Y | N | N | N | NA | Y | N | N | NA | NA | N |  |
| Albikawi et al. | Y | Y | Y | Y | Y | N | NA | N | Y | N | Y | NA | NA | Y | **Good** |
| Alrajeh et al. | Y | Y | N | Y | N | N | NA | N | Y | N | Y | NA | NA | Y |  |
| Cénat et al. | Y | N | CD | Y | N | N | N | NA | Y | N | Y | NA | NA | Y |  |
| Chu et al. | Y | Y | CD | Y | Y | N | NA | N | Y | N | Y | NA | NA | N |  |
| Elipe et al. | Y | N | CD | Y | N | N | N | NA | Y | N | Y | NA | NA | Y |  |
| Feinstein et al. | Y | N | CD | Y | N | N | N | NA | Y | Y | Y | NA | NA | Y |  |
| Felipe-Castaño et al. | Y | Y | CD | Y | Y | N | N | NA | Y | N | Y | NA | NA | Y |  |
| Giumetti et al. | Y | Y | N | Y | N | N | NA | Y | Y | Y | Y | NA | NA | Y |  |
| Huang et al. | Y | Y | Y | Y | N | N | NA | N | N | N | Y | NA | NA | Y |  |
| Kaur et al. | Y | Y | CD | Y | N | N | NA | N | Y | N | Y | NA | NA | Y |  |
| Khine et al. | Y | Y | Y | Y | N | N | N | NA | Y | N | Y | NA | NA | Y |  |
| Kowalski et al. | Y | N | CD | Y | N | N | N | NA | N | N | Y | NA | NA | N |  |
| Lam et al. | Y | Y | Y | N | N | N | NA | Y | Y | N | Y | NA | NA | Y |  |
| Martínez-Monteagudo et al. | Y | N | CD | Y | N | N | N | NA | Y | N | Y | NA | NA | Y |  |
| Medrano et al. | Y | N | CD | Y | N | N | N | NA | Y | N | Y | NA | NA | Y |  |
| Musharraf et al. | Y | N | CD | Y | N | N | N | NA | Y | N | Y | NA | NA | Y |  |
| Na et al. | Y | Y | CD | Y | N | N | N | NA | Y | N | Y | NA | NA | Y |  |
| Sam et al. | Y | N | CD | Y | N | N | N | NA | Y | N | Y | NA | NA | Y |  |
| Schenk et al. | Y | N | CD | Y | N | N | N | NA | Y | N | Y | NA | NA | Y |  |
| Selkie et al. | Y | Y | CD | Y | N | N | N | NA | Y | N | Y | NA | NA | Y |  |
| Wright et al. | Y | Y | CD | Y | N | N | NA | N | Y | N | Y | NA | NA | Y |  |
| Yubero et al. | Y | N | CD | Y | N | N | N | NA | Y | N | Y | NA | NA | Y |  |
| **Key**:  Y: Yes  N: No  CD: Can not be determined  NA: Not Applicable | | | | | | | | | | | | | | | |

| **Supplementary Table 2: Statistical findings among included studies** | | | |
| --- | --- | --- | --- |
| **Reference** | **Factors** | **Statistical Measure** | **Results** |
| **Albikawi et al.** | Self-esteem | Odds ratio | Cyberbully victims were less likely to have higher self-esteem  (aOR = 0.840 [95% CI: 0.740-1.260]; p = 0.748) versus those that had not  experienced cyberbullying. |
|  |  | Bivariate (Pearson) correlation | Weakly significant negative correlation between cybervictimization and  self-esteem (*r* = -0.23; p < 0.001). |
|  | Depression | Odds ratio | Cyberbully victims were not more likely to have experienced depression  (aOR = 0.940 [95% CI: 0.810-0.920]; p < 0.001) versus those that had not  experienced cyberbullying. |
|  |  | Bivariate (Pearson) correlation | Weakly significant negative correlation between cybervictimization and  depression (*r* = -0.33; p < 0.001). |
|  | Internet addiction | Odds ratio | Cyberbully victims were more likely to have experienced internet addiction (aOR = 1.027 [95% CI: 1.010-1.042]; p < 0.001) versus those that had not  experienced cyberbullying. |
|  |  | Bivariate (Pearson) correlation | Moderately significant positive correlation between cybervictimization and  internet addiction (*r* = 0.50; p < 0.001). |
|  | Anxiety | Odds ratio | Cyberbully victims were more likely to have experienced anxiety  (aOR = 1.042 [95% CI: 1.030-1.066]; p < 0.001) versus those that had not  experienced cyberbullying. |
|  |  | Bivariate (Pearson) correlation | Weakly significant positive correlation between cybervictimization and  anxiety (*r* = 0.37; p < 0.001). |
| **Alrajeh et al.** | Depressive symptoms | Odds ratio | Cyberbully victims were more likely to have experienced depression  (aOR = 2.497 [95% CI: 1.704-3.659]; p < 0.001) versus those that had not  experienced cyberbullying. |
|  |  | Bivariate (Pearson) correlation | Weakly significant positive correlation between cybervictimization and  depression (*r* = 0.385; p < 0.01). |
| **Anis-ul-Haque et al.** | Stress | Prevalence | Prevalence of following factors in response to cybervictimization: • Stress: 62.2% • Anxiety: 84.1% • Depression: 73.8% |
|  | Anxiety |  |  |
|  | Depression |  |  |
|  | Well-being | MANOVA | Individuals with cyberbullying experiences had significantly poorer well-being versus those without (F[df] = 2.84 [3.497], p < 0.05). Amongst those with cyberbullying experience, cybervictims had the lowest well-being score (M = 44.36). |
| **Arafa et al.** | Anger | Prevalence | Prevalence of following factors in response to the worst cyberbullying incidents experienced by students within the previous 6 months: • Anger: 63.1% • Hatred: 23.2% • Sorrow: 22.6% • Fear: 16.0% • Disappointment: 14% • Loneliness: 36% |
|  | Hatred |  |  |
|  | Sorrow |  |  |
|  | Fear |  |  |
|  | Disappointment |  |  |
|  | Loneliness |  |  |
| **Beran et al.** | Anger | Prevalence | Prevalence of following factors among the 351 participants who reported being cyber-harassed at least once or twice in university: • Angry: 31.6%  • Hurt: 21.4% • Embarrassed: 20.2% • Anxious: 18.2% • Fear: 12.8% • Crying: 12.5% • Self-blame: 9.1% • Lack of Concentration: 17.9% • Low Achievement: 9.9% • Absenteeism: 7.6% |
|  | Hurt |  |  |
|  | Embarrassed |  |  |
|  | Anxious |  |  |
|  | Fear |  |  |
|  | Crying |  |  |
|  | Self-blame |  |  |
|  | Lack of concentration |  |  |
|  | Low achievement |  |  |
|  | Absenteeism |  |  |
| **Cenat et al.** | Psychological distress | Unstandardized regression coefficient | Weakly significant positive correlation between cybervictimization and psychological distress. (β = 0.330; SE = 0.051; p = 0.00). |
|  | Suicidality |  | Insignificant correlation between cybervictimization and suicidality (β = 0.036; SE = 0.023; p = 0.122). |
| **Chu et al.** | Core self-evaluation | Bivariate (Pearson) correlation | Weakly significant negative correlation between cybervictimization and  core-self-evaluation (*r* = -0.164; p < 0.001). |
|  |  | Unstandardized regression coefficient | Weakly significant negative correlation between cybervictimization and  core-self-evaluation. (β = -0.166; SE = 0.026; p < 0.001). |
|  | Depression | Bivariate (Pearson) correlation | Weakly significant positive correlation between cybervictimization and  depression (*r* = 0.248; p < 0.001). |
|  |  | Unstandardized regression coefficient | Weakly significant positive correlation between cybervictimization and  depression. (β = 0.244; SE = 0.025; p < 0.001). |
|  | Suicidal ideation | Bivariate (Pearson) correlation | Weakly significant positive correlation between cybervictimization and  suicidal ideation (*r* = 0.287; p < 0.001). |
|  |  | Unstandardized regression coefficient | Weakly significant positive correlation between cybervictimization and  suicidal ideation. (β = 0.282; SE = 0.028; p < 0.001). |
| **Elipe et al** | Invigoration | Spearman correlation | Insignificant correlation between cybervictimization and invigoration (*r* = 0.06). |
|  | Annoyance |  | Insignificant correlation between cybervictimization and annoyance (*r* = 0.01). |
|  | Dejection |  | Insignificant correlation between cybervictimization and dejection (*r* = -0.05). |
| **Faucher et al.** | Mental health issues (anxiety, depression, emotional outbursts, etc.) | Prevalence | Prevalence of following factors in response to cybervictimization: • Mental Health Issues: 42% • Suicidal Thoughts: 14% |
|  | Suicidal thoughts |  |  |
| **Feinstein et al.** | Depressive symptoms | T-test | Victims of cyberbullying had significantly greater depressive symptoms versus control participants at both T1 & T2: • T1: 8.32 ± 9.00 vs 6.56 ± 8.44; t= -2.25; p < 0.05 • T2: 9.43 ± 8.75 vs 7.05 ± 8.57; t= -3.04; p < 0.01 |
|  |  | Linear regression | Cyberbully victims were more likely to suffer from depression at T2. (B = 1.21; p < 0.05) |
|  | Rumination | T-test | Victims of cyberbullying had significantly greater rumination versus control participants at both T1 & T2: - T1: 53.29 ± 14.28 vs 48.42 ± 14.57; t = -3.71; p < 0.001 [two-tailed] -T2: 51.73 ± 14.65 vs 46.27 ± 15.13; t = -4.02; p < 0.001 [two-tailed] |
|  |  | Linear regression | Cyberbully victims were more likely to suffer from rumination at T2. (B = 2.23; p < 0.05) |
| **Felipe-Castaño et al.** | Depression | Bivariate (Pearson) correlation | Weakly significant positive correlation between cybervictimization and depression (*r* = 0.270; p < 0.001). |
|  | Hostility |  | Weakly significant positive correlation between cybervictimization and hostility (*r* = 0.340; p < 0.001). |
|  | Interpersonal sensitivity |  | Weakly significant positive correlation between cybervictimization and interpersonal sensitivity (*r* = 0.292; p < 0.001). |
|  | Anxiety |  | Weakly significant positive correlation between cybervictimization and anxiety (*r* = 0.335; p < 0.001). |
|  | Psychoticism |  | Weakly significant positive correlation between cybervictimization and psychoticism (*r* = 0.356; p < 0.001). |
|  | Obsession-compulsion |  | Weakly significant positive correlation between cybervictimization and obsession-compulsion (*r* = 0.249; p < 0.001). |
|  | Phobic anxiety |  | Weakly significant positive correlation between cybervictimization and phobic anxiety (*r* = 0.298; p < 0.001). |
|  | Paranoid ideation |  | Weakly significant positive correlation between cybervictimization and paranoid ideation (*r* = 0.340; p < 0.001). |
| **Giumetti et al.** | Depression | Standardized Beta Coefficients | Significant positive association between cybervictimization and depression (B= 0.36, 95% CI: 0.20-0.53) |
|  | Anxiety |  | Significant positive association between cybervictimization and anxiety (B = 0.38, 95% CI: 0.21-0.54) |
|  | Helping behavior |  | Significant positive association between cybervictimization and helping behavior (B = 0.32, 95% CI: 0.17-0.47) |
|  | Alcohol use |  | Insignificant association between cybervictimization and alcohol use (B = -0.02, 95% CI: -0.13 - 0.10) |
| **Huang et al.** | Self-esteem | T-test | Insignificant difference between victims and non-victims of cyberbullying in social media in overall self-esteem. (30.13 ± 4.81 vs. 30.42 ± 4.59; t = -0.0935; p = 0.350) |
|  |  |  | Insignificant difference between victims and non-victims of cyberbullying in online games in overall self-esteem. (30.37 ± 4.89 vs. 30.51 ± 4.57; t = 1.355; p = 0.182) |
|  | Internet/social media addiction | T-test | Victims of cyberbullying felt significantly greater internet addiction in social media (44.29 ± 10.31 vs. 41.27 ± 10.01; t = 4.442; p < 0.001) than non-victims. |
|  |  |  | Victims of cyberbullying felt significantly greater internet addiction in online gaming (43.59 ± 10.55 vs. 41.92 ± 9.46; t = 1.991; p < 0.047) than non-victims. |
|  |  | Odds ratio | Cyberbully victims in social media were more likely to have experienced internet addiction (aOR = 1.025 [95% CI: 1.010-1.040]; p = 0.001) versus those that had not  experienced cyberbullying. |
|  |  |  | Cyberbully victims in online gaming were more likely to have experienced internet addiction (aOR = 1.018 [95% CI: 1.001-1.037]; p = 0.046) versus those that had not  experienced cyberbullying. |
|  | Anxiety | T-test | Victims of cyberbullying felt significantly greater anxiety in social media (42.04 ± 10.51 vs. 38.00 ± 7.53; t = 6.647; p < 0.001) than non-victims. |
|  |  |  | Insignificant difference between victims and non-victims of cyberbullying in online games in overall anxiety. (40.38 ± 9.72 vs. 39.34 ± 8.83; t = -0.347; p = 0.729) |
|  |  | Odds ratio | Cyberbully victims in social media were more likely to have experienced anxiety (aOR = 1.048 [95% CI: 1.030-1.066]; p < 0.001) versus those that had not  experienced cyberbullying. |
| **Kaur et al.** | Depression | Odds ratio | Cyberbully victims in social media were more likely to have experienced depression (aOR = 2.50 [95% CI: 1.23-5.08]; p < 0.05) versus those that had not  experienced cyberbullying. |
|  | Anxiety |  | Cyberbully victims in social media were more likely to have experienced anxiety (aOR = 2.38 [95% CI: 1.29-4.40]; p < 0.01) versus those that had not  experienced cyberbullying. |
|  | Stress |  | Cyberbully victims in social media were more likely to have experienced stress (aOR = 1.21 [95% CI: 1.12-1.32]; p < 0.001) versus those that had not  experienced cyberbullying. |
| **Khine et al.** | Lack of concentration | Prevalence | Prevalence of following factors following the cyberbullying incident: - Lack of concentration: 23.6% |
|  |  | Odds ratio | Cyberbully victims were more likely to have experienced a lack of concentration (aOR = 3.96 [95% CI: 1.72-9.11]; p = 0.001) versus those that had not experienced cyberbullying. |
|  | Suicidal ideation | Prevalence | Prevalence of following factors following the cyberbullying incident: • Suicidal ideation: 6.0% |
|  |  | Odds ratio | Cyberbully victims were not more likely to have experienced suicidal ideation (aOR = 3.76 [95% CI: 0.44-32.22]; p = 0.227) versus those that had not experienced cyberbullying. |
|  | Substance abuse | Prevalence | Prevalence of following factors following the cyberbullying incident: • Substance abuse: 18.7% |
|  |  | Odds ratio | Cyberbully victims were more likely to have experienced substance abuse (aOR = 2.37 [95% CI: 1.02-5.49]; p = 0.044) versus those that had not experienced cyberbullying. |
| **Kowalski et al.** | Scared | Bivariate (Pearson) correlation | Moderately significant correlation between cybervictimization and feeling scared (*r* = 0.46; p < 0.01). |
|  | Ashamed |  | Weakly significant correlation between cybervictimization and feeling ashamed (*r* = 0.38; p < 0.05). |
|  | Embarrassed |  | Moderately significant correlation between cybervictimization and feeling embarrassed (*r* = 0.47; p < 0.01). |
|  | Helpless |  | Weakly significant correlation between cybervictimization and feeling helpless (*r* = 0.30; p < 0.06). |
|  | Burnout |  | Weakly significant correlation between cybervictimization and burnout (*r* = 0.25; p < 0.01). |
|  | Irritation | T-test | Victims of cyberbullying felt significantly greater irritation (t = 3.04; p < 0.01) than non-victims. |
|  | Anger |  | Victims of cyberbullying felt significantly greater anger (t = 2.15; p <0.05) than non-victims. |
| **Kraft et al.** | Anger | Prevalence | Prevalence of following factors in response to cybervictimization: • Anger: 72% • Frustration: 63% • Upset: 52% • Hurt: 48% • Humiliated: 39% • Distressed: 37% • Sad: 35%  • Depression: 15% • Suicidal thoughts: 2% |
|  | Frustration |  |  |
|  | Upset |  |  |
|  | Hurt |  |  |
|  | Humiliated |  |  |
|  | Distressed |  |  |
|  | Sadness |  |  |
|  | Depression |  |  |
|  | Suicidal thoughts |  |  |
| **Lai et al.** | Overly sensitive to surroundings | Prevalence | Prevalence of following factors in response to cybervictimization: • Overly sensitive to surroundings: 49.4% • Emotional changes: 44.7% • Insecure feelings when surfing the internet: 38.1% • Nervous when receiving messages/emails: 29.6% • Behavioral changes: 35.1% • Avoid mixing with family/friends/crowd: 26.8% • Sleep disorders: 25.7%  • Avoid using computer/mobile phones: 17.0% • Changes in appetite: 17.0% • Suicide attempts: 1.5% |
|  | Emotional changes |  |  |
|  | Insecure feelings when surfing the Internet |  |  |
|  | Nervous when receiving messages/emails |  |  |
|  | Behavioral changes |  |  |
|  | Avoid mixing with  family/friends/crowds |  |  |
|  | Sleep disorders |  |  |
|  | Avoid using computer/ mobile phones |  |  |
|  | Changes in appetite |  |  |
|  | Suicide attempts |  |  |
| **Lam et al.** | Anxiety | Bivariate (Pearson) Correlation | Weakly significant positive correlation between cybervictimization and anxiety (*r* = 0.136; p < 0.01). |
|  | Social skills |  | Insignificant correlation between cybervictimization and social skills. |
| **Lee J** | Depressive symptoms | Bivariate (Pearson) correlation | Weakly significant positive correlation between cybervictimization and depressive symptoms (*r* = 0.23; M[SD] = 2.02 [0.65]; p < 0.01). |
|  | Anxiety symptoms |  | Weakly significant positive correlation between cybervictimization and anxiety symptoms (*r*= 0.27; M[SD] = 1.57 [0.57]; p < 0.01). |
| **Lindsay et al.** | Depression | T-test | Victims of online harassment had significantly greater depression (t = − 2.27 p < 0.05). |
|  |  | Prevalence | Prevalence of the following factors in males and females respectively: • Depressed: 24% and 38% |
|  | Anxiety | T-test | Victims of online harassment had significantly greater anxiety (t = − 2.27 p < 0.05). |
|  |  | Prevalence | Prevalence of the following factors in males and females respectively: • Anxious: 27% and 40% |
| **Martinez Monteguado et al.** | Depression | Prevalence | Prevalence of the following factors in victims of cyberbullying: • Depression: 68.1% |
|  |  | Odds ratio | Cyberbully victims were more likely to have experienced depression (cOR = 1.15 [95% CI: 1.06-1.25]; p = 0.000) versus those that had not experienced cyberbullying. |
|  | Anxiety | Prevalence | Prevalence of the following factors in victims of cyberbullying: • Anxiety: 72.2% |
|  |  | Odds ratio | Cyberbully victims were more likely to have experienced anxiety  cOR = 1.25 [95% CI: 1.12-1.39]; p = 0.000) versus those that had not experienced Cyberbullying. |
|  | Stress | Prevalence | Prevalence of the following factors in victims of cyberbullying: • Stress: 75.2% |
|  |  | Odds ratio | Cyberbully victims were more likely to have experienced anxiety (cOR = 1.43 [95% CI: 1.24-1.64]; p = 0.000) versus those that had not experienced cyberbullying. |
|  | Suicidal thinking | Odds ratio | Cyberbully victims were more likely to have experienced suicidal thinking (cOR = 1.05 [95% CI: 1.01-1.11]; p = 0.000) versus those that had not experienced cyberbullying. |
| **Medrano et al.** | Depression | Bivariate (Pearson) correlation | Weakly significant positive correlation between cybervictimization and depression (*r*= 0.23; M[SD] = 1.89 (0.85); p < .001). |
|  | Suicidal ideation |  | Weakly significant positive correlation between cybervictimization and suicidal ideation. (*r*= 0.37; M [SD] = 1.47 [0.89]; p < .001). |
| **Musharraf et al.** | Depression | Bivariate (Pearson) correlation | Weakly significant positive correlation between cybervictimization and depression (*r*= 0.21; M[SD]= 7.16 [4.78]; p < .01). |
|  |  | Logistical regression | Cyberbully victims were more likely to suffer from depression. (B = 3.22; p < 0.01) |
|  | Anxiety | Bivariate (Pearson) correlation | Weakly significant positive correlation between cybervictimization and anxiety (*r* = 0.29; M[SD] = 7.74 [4.44]; p < .01). |
|  |  | Logistical regression | Cyberbully victims were more likely to suffer from anxiety. (B = 0.28; p < 0.01) |
|  | Stress | Bivariate (Pearson) correlation | Weakly significant positive correlation between cybervictimization and stress (*r*= 0.24; M[SD] = 8.24 [4.58]; p < .01). |
|  |  | Logistical regression | Cyberbully victims were more likely to suffer from stress. (B = 0.24; p < 0.01) |
|  | Well-being | Bivariate (Pearson) correlation | Very weakly significant negative correlation between cybervictimization and well-being (*r* = -0.11; M [SD] = 45.84 [12.22]; p < .05). |
|  |  | Logistical regression | Cyberbully victims were not more likely to have poorer well-being. (B = -0.10; p > 0.01) |
| **Na et al.** | Depression | Bivariate (Pearson) correlation | Weakly significant positive correlation between cybervictimization and depression (*r* = 0.23; M[SD] = 12.74 [11.04]; p < .05). |
|  |  | Linear regression | Increased frequency of cyberbully victimization was not more likely to lead to depression. (B = 0.147; p> 0.05) |
|  | Anxiety | Bivariate (Pearson) correlation | Weakly significant positive correlation between cybervictimization and anxiety (*r* = 0.23; M[SD] = 12.68[9.82]; p < .05). |
|  |  | Linear regression | Increased frequency of cyberbully victimization was not more likely to lead to anxiety. (B = 0.252; p > 0.05) |
|  | Self-esteem | Bivariate (Pearson) correlation | Weakly significant negative correlation between cybervictimization and self-esteem (*r* = -0.39; M[SD] = 20.35 [5.38]; p < .05). |
|  |  | Linear regression | Increased frequency of cyberbully victimization was more likely to lead to poorer self-esteem. (B = -0.399; p < 0.05) |
| **Sam et al.** | Psychological symptoms | T-test | Victims had significantly higher scores on the overall psychological symptoms scale as compared to non-victims. (2.30 ± 0.59 vs. 1.99 ± 0.52; t (474) = 2.74; p < 0.01) |
|  | Anxiety |  | Insignificant difference between victims and non-victims on the overall anxiety scale. (2.36 ± 0.78 vs. 2.08 ± 0.61; t (474) = 1.93; p >0.05) |
|  | Depressive symptoms |  | Insignificant difference between victims and non-victims on the overall depressive symptoms scale. (2.29 ± 0.77 vs. 2.03 ± 0.66; t (474) = 1.81; p >0.05) |
|  | Self-Esteem |  | Insignificant difference between victims and non-victims on the overall self-esteem scale. (3.96 ± 0.73 vs. 3.90 ± .84); t (474) = 0.42; p >0.05) |
| **Schenk et al.** | OCD | MANOVA | Insignificant relationship between cybervictimization and OCD (M[SD] = 59.42[12.18]; F = 0.92; p = 0.340; Partial η2 = 0.007). |
|  | Interpersonal sensitivity |  | Insignificant relationship between cybervictimization and interpersonal sensitivity (M[SD] = 58.17[13.16]; F = 6.62; p = 0.011; Partial η2 = 0.047). |
|  | Depression |  | Victims were significantly elevated on depression subscales (M[SD] = 61.13[10.91]; F = 9.90; p = 0.002; Partial η2 = 0.069). |
|  | Anxiety |  | Victims were significantly elevated on anxiety subscales (M[SD] = 57.20[13.34]; F = 9.11; p = 0.003; Partial η2 = 0.064). |
|  | Hostility |  | Insignificant findings between cybervictimization and hostility. (M[SD]=  57.23[10.70]; F = 6.65 p = 0.011; Partial η2 = 0.047). |
|  | Phobic anxiety |  | Victims were significantly elevated on phobic anxiety subscales (M[SD] = 56.29[11.92]; F = 8.66; p = 0.004; Partial η2 = 0.061). |
|  | Paranoid |  | Victims were significantly elevated on paranoia subscales (M[SD] = 56.77[12.541]; F = 9.90; p = 0.001; Partial η2 = 0.083). |
|  | Psychotic |  | Insignificant findings between cybervictimization and psychotic subscales (M[SD]= 58.41[14.30]; F = 6.54; p = 0.012; Partial η2 = 0.047). |
|  | Suicidal behaviours | Prevalence | Prevalence of factors in victims vs controls, respectively: •Suicide attempts: 5.7% vs 0.0% •Suicidal planning: 15.9% vs 10.1% • Suicidal ideation: 29.0% vs 18.8% |
|  |  | Chi-squared | Frequency of suicidal planning/attempts was significantly greater between control participants and victims, χ2 (3, n = 138) = 9.09, p = .028. Frequency of suicidal ideation was also significantly greater in this sample, χ2 (2, n = 138) = 7.38, p = .025 |
|  |  | T-Test | Victims had a significantly greater total suicide score for overall suicidal behavior compared to controls. (2.39 ± 3.52 vs 1.00 ± 2.00; t = -2.85; p = 0.005) |
| **Selkie et al.** | Depression | Odds ratio | Cyberbully victims were not more likely to have experienced depression (aOR = 2.1 [95% CI: [0.9–4.9]; p = 0.07) when compared with those who had not experienced cyberbullying. |
|  | Alcohol abuse |  | Cyberbully victims were not more likely to have experienced alcohol abuse (aOR = 1.1 95% CI: [0.5–2.3]; p = 0.76) when compared with those who had not experienced cyberbullying. |
| **Snaychuk et al.** | Self-esteem | T-Test | Victims of TFSV had significantly higher scores on the overall self-esteem scale as compared to non-victims. (15.60 ± 5.801 vs. 20.66 ± 4.856; t (110) = -4.87; p < 0.001) |
|  | Social support |  | Insignificant difference between victims and non-victims of TFSV on the overall social support scale (58.41 ± 15.367 vs. 63.69 ±17.085; t (115) = -1.75; p = 0.082) |
|  | Perceived control |  | Victims of TFSV had significantly lower scores on the overall perceived control scale as compared to non-victims. (24.16 ± 5.556 vs. 28.86 ± 3.98; t (112) = -5.29; p < 0.001) |
|  | Depression |  | Victims of TFSV had significantly higher scores on the overall depression scale as compared to non-victims. (19.57 ± 13.530 vs. 6.64 ± 5.989; t (88) = 6.63; p < 0.001) |
| **Wright et al.** | Depression | Unstandardized regression coefficient | Weakly significant positive correlation between cybervictimization and  psychological distress. (β = 0.29; SE = 0.10; p < 0.001). |
| **Yubero et al.** | Self-esteem | T-test | Victims had significantly lower self-esteem  (t = 2.27; p < 0.05) versus non-victims. |
|  |  | Bivariate (Pearson) correlation | Very weakly significant negative correlation between cybervictimization and self-esteem (*r* = -0.152; p < .05). |
|  |  | Odds ratio | Cyberbully victims were not more likely to have lower self-esteem (cOR = 0.76 [95% CI: [0.23–2.48]) when compared with those who had not experienced cyberbullying. |
|  | Loneliness | T-test | Victims had significantly greater loneliness t = −2.54; p < .01) versus non-victims. |
|  |  | Bivariate (Pearson) correlation | Very weakly significant positive correlation between cybervictimization and loneliness (β = 0.149; p < .05). |
|  |  | Odds ratio | Cyberbully victims were not more likely to have more loneliness (cOR = 1.37 [95% CI: [0.46–4.10]) when compared with those who had not experienced cyberbullying. |
|  | Perceived acceptance by peers (PAP) | T-test | Victims had significantly lower PAP t = −2.70; p < .05) versus non-victims. |
|  |  | Bivariate (Pearson) correlation | Weakly significant negative correlation between cybervictimization and PAP (*r* = -0.278; p < .01). |
|  |  | Odds ratio | Cyberbully victims were more likely to have lower PAP (cOR = 3.57 [95% CI: 1.29-9.87]; p < 0.001) versus those that had not experienced cyberbullying. |
| **Zalaquett et al.** | Anger | Prevalence | Prevalence of following factors in participants who reported experiencing cyberbullying: • Anger: 45% • Sad: 41% • Stressed: 32% • Loss of productivity: 9% |
|  | Sadness |  |  |
|  | Stressed |  |  |
|  | Loss of productivity |  |  |
| **Key**:  TFSV: Technology facilitated sexual violence  MANOVA: Multivariate analysis of variance M: Mean SD: Standard deviation SE: Standard error cOR: Crude odds ratio aOR: Adjusted odds ratio | | | |
