# Supplementary figures and images for: The impact of cyberbullying on mental health outcomes amongst university students: A systematic review
Source: PLOS Ment Health. 2024 Nov 13;1(6):e0000166. doi: 10.1371/journal.pmen.0000166 (PMC12798282; doi:10.1371/journal.pmen.0000166)

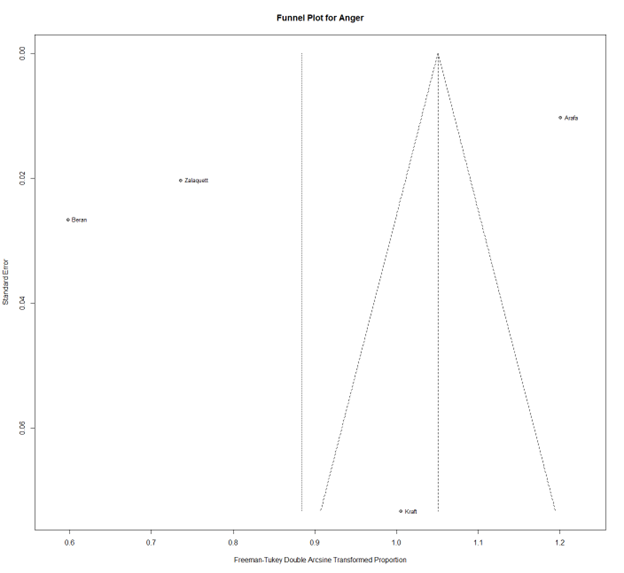

Supplement: S1 Fig — (TIF) [file pmen.0000166.s005.tif]

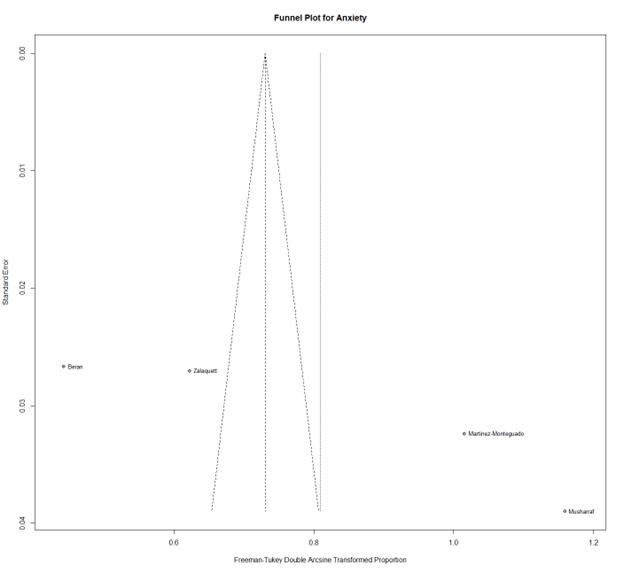

Supplement: S2 Fig — (TIF) [file pmen.0000166.s006.tif]

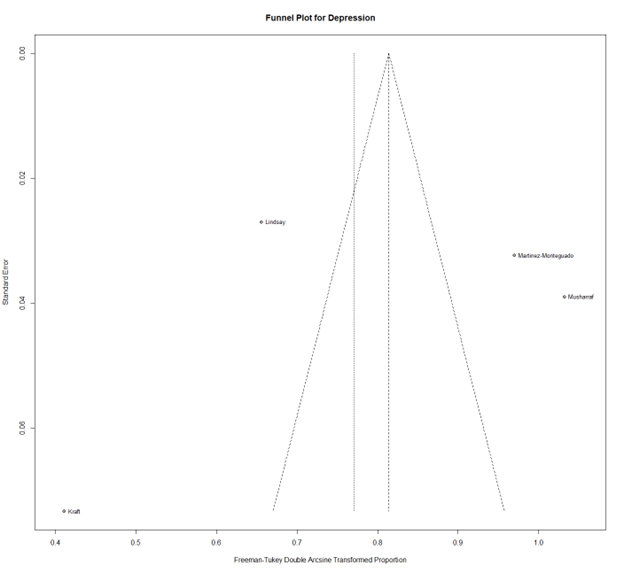

Supplement: S3 Fig — (TIF) [file pmen.0000166.s007.tif]

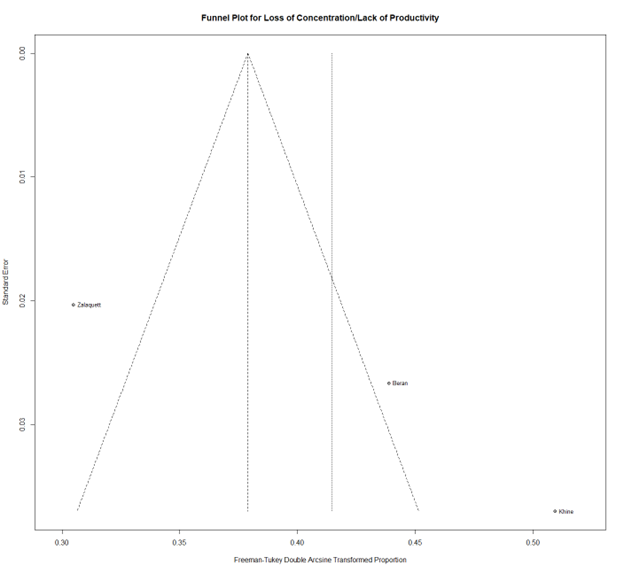

Supplement: S4 Fig — (TIF) [file pmen.0000166.s008.tif]

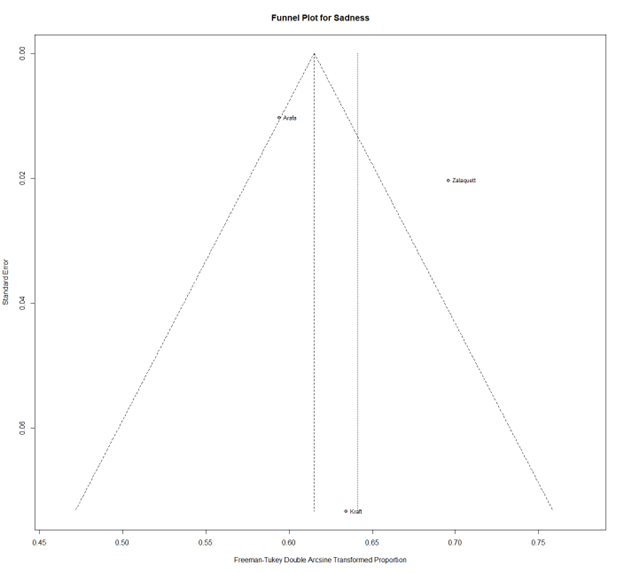

Supplement: S5 Fig — (TIF) [file pmen.0000166.s009.tif]

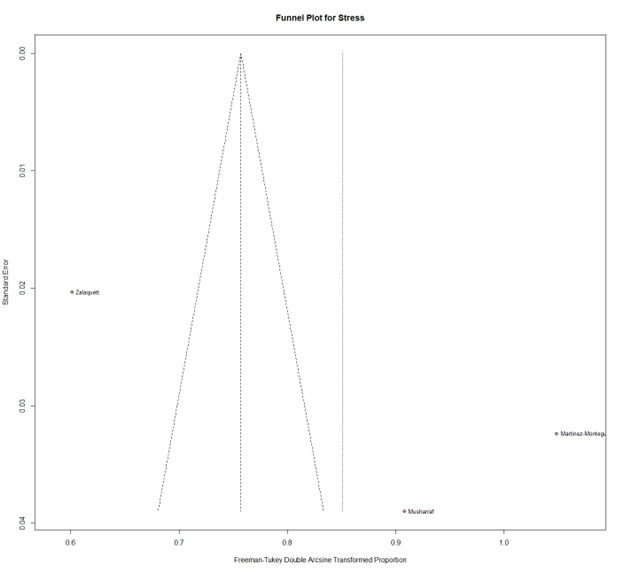

Supplement: S6 Fig — (TIF) [file pmen.0000166.s010.tif]

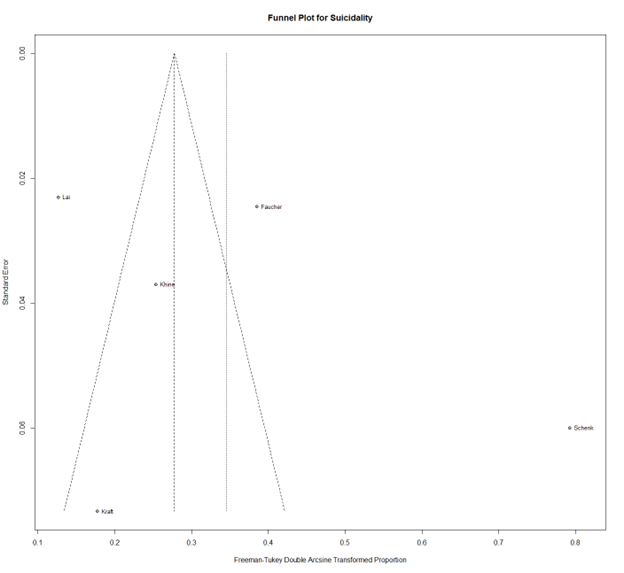

Supplement: S7 Fig — (TIF) [file pmen.0000166.s011.tif]
