# Supplementary material for: The impact of cyberbullying on mental health outcomes amongst university students: A systematic review
Source: PLOS Ment Health. 2024 Nov 13;1(6):e0000166. doi: 10.1371/journal.pmen.0000166 (PMC12798282; doi:10.1371/journal.pmen.0000166)
Supplement: Attachment — Submitted filename: Response to Reviewers..docx [file pmen.0000166.s012.docx]

Wenjie Duan, Ph.D.
Academic Editor
PLOS Mental Health
June 23, 2024

Re: PLOS Mental Health Decision: Revision required [PMEN-D-24-00118]

Dear Dr. Duan,

Thank you for reviewing our manuscript entitled “**The Impact of Cyberbullying on Mental Health Outcomes amongst University Students: A Systematic Review**”

We are pleased that our manuscript was favorably reviewed and was found to be potentially acceptable for publication pending revisions.

We thank the reviewers for their valuable insight and comments as these serve to further strengthen our manuscript.

As requested, we have provided a point-by-point response to each of the reviewer’s comments with relevant changes made to the manuscript.

**Reviewers:**

Reviewer 1:

*The manuscript titled is a comprehensive effort to synthesize the existing research on the psychological effects of cyberbullying among university students. However, there are several areas where the study could be improved or where limitations should be acknowledged more explicitly*

We thank the Reviewer for their review and for suggesting ways to further improve our work.

Suggestions:

*1.* *The manuscript highlights the lack of a uniform definition of cyberbullying across studies, which may have led to variability in findings. This is a critical limitation as it affects the comparability of study results and may inflate or deflate the prevalence rates reported.*

Thank you for your feedback. We agree that this heterogeneity of definitions across articles limits the comparability of study results. As such, we have acknowledged this in our **Discussion** Paragraph 6, Page 13: “Firstly, the tools and definitions used to define and measure cyberbullying and mental health outcomes differed across studies and precluded statistical pooling of data. As such, there is considerable heterogeneity across the dataset thus limiting the generalizability of the data and potentially underestimating or overestimating of the prevalences of mental health outcomes. This heterogeneity further signifies the need to establish a uniform framework to identify and counteract cybervictimization. Despite this limitation, however, our study is the first systematic review to evaluate the impact of cyberbullying in developing mental health outcomes, thus highlighting a critical avenue for further research”.

*2. Many of the studies included in the review are cross-sectional, which limits the ability to infer causality between cyberbullying and mental health outcomes. The review could benefit from a discussion on the need for longitudinal studies to better understand the causal pathways.*

Thank you for highlighting this point. We agree that the study designs of papers included in this review limits inferences that may be drawn regarding cyberbullying and mental health outcomes. We have incorporated this in our **Discussion** Paragraph 5, Page 12-13: “Similarly, the majority of articles included in this study were of a cross-sectional study design, thus preventing accurate inference of causality in the relationship between cybervictimization and mental health outcomes. Due to these limitations, it is possible that the conclusions drawn within this review may be over or under-estimated. As such, it is imperative that further research is conducted emphasizing a longitudinal and robust approach, thus allowing for a more accurate assessment of mental health outcomes”.

*3. While the manuscript notes that the majority of articles were of good quality, there are still several that were of fair or poor quality. This could potentially bias the overall findings of the review. A more thorough discussion on how these quality issues might impact the review's conclusions would be beneficial.*

Thank you for bringing this point to light. We have added a discussion regarding the potential for quality issues to impact the study conclusions in our **Discussion**, Paragraph 6, Page 13-14: “Secondly, only 22 out of the 32 included articles were deemed to be of good quality, with the rest being fair or poor, with such articles failing to validate their study questionnaires. Such lack of validation may result in a lower quality of evidence, due to reduced reliability of results and a potential increase in study bias preventing inference of study outcomes. These findings were manifested in our review, where ten articles were found to be fair or poor, thus increasing the potential for heterogeneity and reduced validity of inferences”.

*4. The studies included span a range of geographic and cultural contexts, which can influence both the experience of cyberbullying and its impact on mental health. The review could strengthen its analysis by more deeply exploring how cultural differences affect the generalizability of the findings.*

Thank you for pointing this out. We have incorporated a discussion of the cultural contexts relating to the development of mental health outcomes in our **Discussion**, Paragraph 4, Page 11: “Furthermore, literature has demonstrated the impact of cultural contexts on the perception and response to bullying, where western cultures were found to be more forthcoming and receptive towards the entity. These results are not dissimilar to our findings, where certain outcomes such as anger and depressive symptoms were seen to be higher amongst such regions. Thus, it may be possible that increased awareness and openness to cybervictimization may enable improved coping and reduced development of outcomes in certain geographic regions, however, further research is necessitated to warrant this assumption”.

*5. The review does not discuss the possibility of publication bias, where studies with significant findings are more likely to be published than those with null results. Addressing this could involve a statistical assessment of publication bias to ensure the robustness of the review's conclusions.*

Thank you for highlighting this point. We have updated the manuscript to reflect analysis of publication bias using in the **Methods, Results and Discussion** section: “Publication bias was assessed using R. version 4.3.0. Outcomes where prevalences was available for at least three articles were included. To evaluate outcomes, the Free-man Turkey double arcsine transformation was utilized, with subsequent generation of funnel plots (**S4 Supplementary Figures**). Funnel plots were then tested for statistical evidence of bias using the Begg funnel plot test.”, “A total of seven outcomes (anger, anxiety, depression, lack of concentration/loss of productivity, sadness, stress, and suicidality) across eleven articles were analyzed for potential publication bias. Strong evidence of publication bias was found when evaluating anxiety (**p = 0.0415**). The remainder of outcomes demonstrated no study publication bias (**S4 Supplementary Figures**).”, and “Our results demonstrated that publication bias was only found in one outcome (anxiety), however, due to the limited number of articles evaluating outcomes, it is possible that further publication bias is present. Thus, it is imperative that great nuance is employed when interpreting our results, however, the studies included in this review were conducted across a wide range of settings, which increases the generalizability of our conclusions”.

*6. While the review recommends interventions, there is limited discussion on the effectiveness of existing cyberbullying interventions, particularly those targeted at university students. An analysis of intervention studies could provide valuable insights into what works and what doesn’t in preventing or mitigating the effects of cyberbullying.*

Thank you for pointing this out. We agree that a more thorough discussion on pre-existing literature regarding interventions at the university level has not been elaborated on in the discussion. We have corrected this in the **Discussion** section, Paragraph 5, Page 12-13, to acknowledge this: “Though interventions have been studied extensively at the adolescent level; there is a significant lack of literature in the setting of higher education. Literature has demonstrated that interventions in the university setting have included interventional videos, zero-tolerance policies, and cyberbullying reporting systems however limited research has been conducted on the effectiveness of such interventions. Furthermore, barriers towards intervention access are difficult to address, with studies finding university students to be apprehensive when seeking help due to perceptions of cyberbullying as a “juvenile” issue. As such, it is imperative that further research is conducted to determine systemic barriers towards intervention access, and the impact of a wider array of interventions in the higher education setting.”

*7. The review mentions that all data are included in the published article, but it does not discuss the availability of raw data for reanalysis or the reproducibility of the results, which are important for verifying and building on research findings.*

Thank you for your feedback. We have updated the manuscript to reflect the availability of raw data for reanalysis and reproducibility on Page 22, under the section titled “**Availability of data and materials**” with the following addition: “The raw data used and analyzed during the current study are available from the corresponding author on reasonable request.”

The authors are extremely appreciative of the Reviewer’s suggestions and believe that they contributed significantly to further strengthening the paper.

Thank you for considering our revised manuscript.

Hussain Maqbool Ahmed Khuwaja, MSc
School of Nursing and Midwifery
Aga Khan University
Karachi 74800, Pakistan
